# On the Treatment of a Peculiar Form of Diarrhœa

**Published:** 1845-12

**Authors:** 


*On the Treatment of a peculiar form of Diarrhœa.*—Dr. Corrigan has called attention to a form of diarrhœa, of frequent occurrence in the upper classes of society. He observes:

"The persons most subject to this complaint are females, of what is termed the leuco-phlegmatic temperament, characterized by weak, flabby, muscular development, and general indolence of constitution. In such persons, the menses may be either profuse, or sparing, in quantity; or they may be regular; but this is of no consequence. Connected with this bowel complaint, you will always find more or less of leucorrhœa, which, on inquiry, you will find to have preceded the diarrhœa: as though it would seem that the mucous membrane of the intestines becomes affected just in the same manner as the epithelium of the vagina; these persons are also affected with "bruit de soufflet" of the heart, which is always audible to themselves, and causes them great anxiety. You will find them also, laboring under catarrh, with copious secretion, accompanied by pain of a neuralgic character, here to-day and gone to-morrow; they get drooping and depressed in spirits; lose all inclination for mixing in their usual society; and then their friends, fearing the occurrence of phthisis, become alarmed at their delicate state of health, and, in great consternation, look for advice. This train of symptoms would appear to be caused by the baneful effects of living much in fashionable life, and by stopping up late of nights in crowded rooms, to enjoy their pleasures. These evils, joined to the already existing one of a constitution habitually delicate, are quite enough to produce the disease in question. For the cure of this I have seen the Pharmacopœia unceasingly ransacked; I have seen the most powerful astringents tried, separately, and in combination with other remedies of the same character; still no good was done by them, while afterward the disease has yielded, and that quickly, under the exhibition of decoctum hæmatoxyli, as recommended by Dr. Abercrombie, in doses of a wine-glassful, three times a day. I had, not long since, a lady under my care, laboring with this distressing affection, in whom it was quickly subdued by this simple remedy.—*Ranking's Half-yearly Abs.*

